# Mechanistic Insight and Management of Diabetic Nephropathy: Recent Progress and Future Perspective

**DOI:** 10.1155/2017/1839809

**Published:** 2017-03-13

**Authors:** Rui Xue, Dingkun Gui, Liyang Zheng, Ruonan Zhai, Feng Wang, Niansong Wang

**Affiliations:** Department of Nephrology, Shanghai Jiao Tong University Affiliated Sixth People's Hospital, Shanghai, China

## Abstract

Diabetic nephropathy (DN) is the most serious microvascular complication of diabetes and the largest single cause of end-stage renal disease (ESRD) in many developed countries. DN is also associated with an increased cardiovascular mortality. It occurs as a result of interaction between both genetic and environmental factors. Hyperglycemia, hypertension, and genetic predisposition are the major risk factors. However, the exact mechanisms of DN are unclear. Despite the benefits derived from strict control of glucose and blood pressure, as well as inhibition of renin-angiotensin-aldosterone system, many patients continue to enter into ESRD. Thus, there is urgent need for improving mechanistic understanding of DN and then developing new and effective therapeutic approaches to delay the progression of DN. This review focuses on recent progress and future perspective about mechanistic insight and management of DN. Some preclinical relevant studies are highlighted and new perspectives of traditional Chinese medicine (TCM) for delaying DN progression are discussed in detail. These findings strengthen the therapeutic rationale for TCM in the treatment of DN and also provide new insights into the development of novel drugs for the prevention of DN. However, feasibility and safety of these therapeutic approaches and the clinical applicability of TCM in human DN need to be further investigated.

## 1. Introduction

Diabetic nephropathy (DN) is a major complication of diabetes and the largest single cause of end-stage renal disease (ESRD) in many developed countries. DN is also associated with an increased cardiovascular mortality. Immunologic derangement is the cornerstone of pathogenesis of CKD [1]. Of course, both primary kidney disease and secondary kidney disease dwell in this process, including DN [2]. DN is the most serious microvascular complication of diabetes and the largest single cause of ESRD in many developed countries. DN is a usual cause of ESRD in regions in which the availability of renal replacement therapy (RRT) is limited [3]. The global prevalence of diabetes is growing rashly and rapidly, especially in developing countries. Furthermore, regardless of the underlying causes of DN, the multitudinous treatments of DN do not apparently reduce its substantial morbidity and mortality and overspent disproportionate heath care expenditure and are conducted as a tremendous socioeconomic burden on society [4]. Therefore, there is urgent need for improving mechanistic understanding of DN and then developing new and effective therapeutic approaches to delay the progression of DN. This review focuses on recent progress and future perspective about mechanistic insight and management of DN.

## 2. The Epidemiology of DN

### 2.1. DN in General

DN is one of the most frequent and severe complications of diabetes mellitus and a worldwide public health problem affecting millions of people. Currently, based on the criteria of the kidney disease, Improving Global Outcomes (KDIGO) Clinical Practice Guideline, DN was diagnosed by renal biopsy or medical history [5]. DN is the largest single cause of ESRD in many developed countries and will continue to be the leading cause of death in diabetes mellitus [6, 7]. According to the data of American Diabetes Association, diabetes mellitus has a major impact on the development of DN. DN occurs in 20% to 40% of all patients with type 2 diabetes mellitus, accounting for approximately 50% of cases in the developed countries [8, 9]. In short, counting on the undiagnosed patients who do not yet realize their condition, the overall prevalence of DN is unoptimistic. Particularly, Table 1 summarizes selected studies that evaluated DN incidences and outcomes in diabetic nephropathy patients and also are related to the topic [10–16]. American Diabetes Association accurately defined diabetes mellitus as “a group of metabolic diseases characterized by hyperglycemia resulting from defects in insulin secretion, insulin action, or both” [17]. And it is dreadful because of the complications including long-term dysfunction and failure of different organs.

### 2.2. DN in China

The prevalence of DN has been dramatically increased in recent decades; especially in China, the adjusted prevalence of diabetes mellitus and prediabetes among Chinese adults is 9.7% and 15.5%, respectively [18, 19]. In China, DN is growing at an alarming rate. A national survey which started by Peking University Institute of Nephrology indicates that China is suffering from afflictions associated with increasingly unhealthy diets and obesity. Besides a rise in the incidence of diabetes, the growth rate of CKD has been surpassed in the United States, although the number of Chinese citizens with CKD dwarfs the number in the United States [20]. The situation of China on the whole is that, owing to the lack of financial and clinical resources, as well as inequalities in access to across all regions and populations, the prevention and management of DN is unfulfilled in the last few decades. But with the advocacies and endeavors of the government and the Chinese Society of Nephrology (CSN) increases, the high efficiency health care system has covered almost all the population, and patient care and medical education in the field of DN have been greatly improved in the past years [21]. The currently available treatment improves the conditions of DN patients but still cannot completely stop the progression of DN to ESRD. Therefore, there is an urgent need for the development of novel therapeutic approaches for the treatment of DN.

## 3. The Pathophysiological Process of DN

DN occurs as a result of interaction between both genetic and environmental factors. Hyperglycemia, hypertension, and genetic predisposition are the major risk factors. The aggravating hyperglycemia is also a high risk factor for the development of microvascular complications [22]. In other words, patients with diabetes mellitus tend to have increased the risk of developing microvascular complications, especially organs with arterioles and microvessels, such as eyes and kidney, which causes horrible retinopathy and nephropathy [23]. The pathogenesis of the microvascular complications of diabetes mellitus is not yet fully understood, but hyperglycemia always acts as an initiating and sustaining factor to continuously damage target tissues and organs. Tissues susceptibility increases as a result of significant microvasculopathy and of interstitial inflammation. Because kidney is an organ with high bioenergetic needs, hyperglycemia with its glucotoxicity makes arterioles and microvessels hyaline degeneration and fibrinoid degeneration which seems to be triggering a vicious cycle and causes damage to renal mitochondria resulting in bioenergetic deficit of renal tubular epithelial cells [24].

Podocyte damage occurs at a relatively early stage in CKD [25]. Podocyte is a terminal differentiated glomerular epithelial cell which locates the outside of the glomerular basement membrane (GBM) and as the final barrier of glomerular filtration barrier, podocyte leads a crucial role in establishing the selective permeability of the glomerular filtration barrier [26, 27]. Severe podocyte injury could appear in DN, especially the fusion of podocyte foot processes (FPs). Such phenomenon may explain why podocyte injury is typically associated with marked proteinuria in DN [28]. Furthermore, in the research of multiphoton excitation fluorescence microscopy of the filtration barrier, they have further visualized a new information on intact podocyte and also provided an additional multiphoton evidence for the glomerular origin of proteinuria by imaging the morphologic and functional consequences of localized podocyte damage using the experimental model of FSGS and localize the areas of podocyte damage [29]. Hence, the concept of podocyte loss or detachment as an important factor in the glomerulosclerotic process in diabetes is well documented. The promise of this new insight is the development of new and effective strategies for the prevention and treatment of DN.

## 4. Prevention and Management of DN

### 4.1. The Present Therapies

The management of diabetes mellitus hinges on “five carriages,” which are health education, diet, exercise, weight control, and drug treatment, respectively, of which the former four therapies are described as nonpharmacological measures. And the pharmacological agents currently approved for treatment of diabetes mellitus include sulfonylureas, metformin, acarbose, and insulin [30]. Nonpharmacological measures are critical to the early stages of diabetes mellitus, but people who often ignore the importance of nonpharmacological measures are often ignored, while pharmacological measures are the most frequently used therapies at this stage and the goal would be to have a drug ameliorate or correct both of these abnormalities in the patient with diabetes mellitus. Fortunately, multitudinous research continues to furnish advanced understanding of the pathophysiology and outcomes of this disease, which could help patients to change views and to control diabetes mellitus better with proper diet, applicable exercise, and advisable weight control [31]. All of these are the principles of management of diabetes; management of DN could be fit for the same principles. Now, the principles of the management of DN could be summarized in three parts: glycemic control, management of proteinuria, and intervention of merging symptoms.

#### 4.1.1. Glycemic Control

There is no doubt that glycemic control plays the most momentous role in management of DN; keeping blood glucose within normal limits is a foundation and prerequisite for the treatment of DN [32]. Several studies have indicated that a close relationship has been established between poor glycemic control and microvascular complications, including DN [33]. And many studies have confirmed that strict glycemic control could generate a beneficial effect to slow down the progression of DN and significantly decrease albuminuria in trials [34]. According to many previous studies, patients with diabetes mellitus have been shown to have defects in sensitivity of target organs and tissues to insulin and to relatively inadequate insulin output [35]. Based on these, there are two ways to correct hyperglycemia: one is increasing sensitivity of target organs and tissues to insulin and another is stimulating insulin secretion, corresponding to the clinical commonly used first-line drugs thiazolidinediones of which representative drug is troglitazone and sulfonylureas, respectively. Troglitazone is an antidiabetic antihyperglycemic agent; tons of research of troglitazone have been studied for more than a decade [36]. As one of the only commonly used drugs, troglitazone exploits tissue sensitization to insulin as the main mechanism of action and exert a furthersome effect on *β*-cells function of pancreatic island [37, 38]. Hence, the risks of diabetic complications could be reduced. Secondly, the sulfonylureas have been regarded as the primary drug for treatment of diabetes mellitus for decades, and the dominating function of sulfonylureas is stimulate insulin secretion by *β*-cells of pancreatic island [39]. Moreover, sulfonylurea therapies could lower the blood glucose levels and raise plasma insulin levels in the untreated patients with diabetes mellitus and postprandial blood glucose levels and low plasma insulin levels [40]. Sulfonylureas augment insulin secretion has no direct actions on insulin sensitivity which is totally different with thiazolidinediones [41]. In addition, there are two types of drugs commonly used in daily clinical aside from thiazolidinediones and sulfonylureas, metformin, and acarbose. They are distinguished very effectively in lowering blood glucose in patients with diabetes mellitus with minimal side-effects; besides, studies have found that metformin also could exert renoprotective properties owing to its multieffects on multiple signaling pathways apart from its use as an antidiabetic drug [42–44]. Taken together, despite the benefits derived from strict control of glucose, many patients continue to enter into ESRD. Thus, there is urgent need for the development of new and effective therapeutic approaches to prevent DN.

#### 4.1.2. Management of Proteinuria

Proteinuria is considered as a hallmark and sensitive marker of DN, which is mainly caused by the severe podocyte injury [45]. But what could we do to deal with proteinuria in clinic now? Conventional angiotensin converting enzyme inhibitor (ACEI) or angiotensin receptor blocker (ARB) is recommended for the management of DN, especially for patients of DN with high blood pressure [46]. They blocked the renin-angiotensin-aldosterone system (RAAS) pathway, which is the most important component in the development and progression of DN [47]. In addition, a breakthrough of emerging evidence shows that the use of mineralocorticoid receptor blockers (MRB) in combination with ACEI or ARBs might have benefits on proteinuria [48]. In conclusion the long-term clinical use of ACEI or ARB has been proven safe and effective but still needs to be used with caution in those with decreased renal function, especially with severe kidney failure.

#### 4.1.3. Intervention of Merging Symptoms

At the same time, many patients of DN still show merging symptoms, such as dyslipidemia and hypertension. Poorly controlled blood glucose levels in patients with DN are associated with dyslipidemia which constitutes an additional risk factor. Dyslipidemia consists of elevated levels of low density lipoprotein cholesterol and low levels of high-density lipoprotein cholesterol and increased levels of apolipoproteins B [49]. In view of this, statins, 3-hydroxy-methylglutaryl coenzyme A (HMG-CoA) reductase inhibitors, are recommended for DN with normal low density lipoprotein levels as well and have a major role in preventing another long-term complications in diabetes mellitus [50]. Fortunately, these abnormalities could also be improved by favorable control of blood glucose levels [51]. Hypertension is a common comorbidity usually following the development of DN, several multiple prospective randomized placebo-controlled trials demonstrate that tight blood pressure control among patients with DN could decrease the rates of macrovascular complications as well as microvascular complications [52]. Given the pathogenesis and clinical symptoms of DN with hypertension, ACEI or ARB therapies could slow nephropathy progression which means that the hallmark of very high proteinuria of DN could have a great relief while restoring kidney function in the meantime [53]. Hopefully, new therapeutic strategies and directions of these present therapies could be explored through our unremitting efforts with the advance in the understanding of DN.

## 5. The Future Therapies of DN

Cutting-edge evidence shows that there are certain trends of the future therapies for DN, including comprehensive exploration of existing medicines, application of molecular biology discoveries, progress in stem cell therapy, and the applicability of traditional Chinese medicine (TCM) in the treatment of DN.

### 5.1. Comprehensive Exploration of Existing Medicines

As mentioned previously, The RAAS pathway is very important in the progression of DN. Several studies indicate that the combination of spironolactone with an ACEI or ARB could significantly mitigate proteinuria compared with the placebo [47, 54]. Furthermore, there are evidences which indicate that lack of a vitamin D receptor (VDR) which is a member of the nuclear receptor superfamily and acts as an ordinary nuclear receptor hormone could directly but not exclusively regulate gene transcription, resulting in the induction or suppression of vitamin D target genes which resulted in an increase in RAAS activity and significant proteinuria [55, 56], which means 1,25(OH)2D3 that exert renal protective effects and might involve suppression of renin gene transcription as well as high glucose-induced angiotensinogen production [57]. By the way, research reported that 1,25(OH)2D3 could reduce the hypercorrection of fibroblast growth factor 23 (FGF23) level which is also a risk factor for the progression of DN and could damage podocyte [58]. These studies strongly supported the recommendation of vitamin D supplementation for the prevention and management of DN.

### 5.2. Application of Molecular Biology Discoveries

Studies performed have discovered several key signaling pathways in succession, such as endoplasmic reticulum (ER) stress, which already have become a therapeutic target of much concern for DN [59]. Besides, there are researches of the glucagon-like peptide-1 (GLP-1) which are secreted by the intestinal enteroendocrine cells in response to ingestion of various nutrients showing that GLP-1 can bind to *β*-cell receptors, stimulate insulin release, and improve glycemic control and then play a protecting role in management of DN [60]. Molecular biology has a great development in decades, especially in the 21st century; in recent years, studies of nuclear factor kB (NF-kB) which has been postulated in many immune systems and inflammation are on fire. NF-*κ*B is a ubiquitous nuclear transcription factor that regulates expression of a large number of genes that are critical for the regulation of inflammation [61]. The concrete mechanisms are that NF-kB promotes the expression of a number of genes involved in inflammation, such as cytokines, and activates apoptosis process, and might be able to translate the therapeutic potentials for DN into reality [62].

### 5.3. Progress in Stem Cell Therapy

Stem cells that are identified nearly half a century ago exhibited great potential for the repair of damaged tissues and organs and provided new hope for a means to change the track of DN [63]. Multiple preclinical studies have demonstrated that the innovative stem cell therapy could also be used in kidney, such as the using of mesenchymal stem cells (MSCs) of late years [64]. MSCs are undifferentiated cells capable of self-renewal and multilineage differentiation. Interestingly, the administration of MSCs could prevent renal injury and promote renal recovery through a series of complex mechanisms and on account of the therapeutic potentials. Therefore, MSCs are being evaluated as a possible member in treatment of DN [65]. Apoptosis of stem cells is likely to promote eventual manifestation of kidney failure in diabetes mellitus [66]. Furthermore, the present study shows reductions in subsets of stem cells in peripheral blood and renal cell preparations of db/db mice which is a model of DN, which indicates that the decrease of stem cells might be a hallmark of DN [66, 67]. MSC transplantation is a promising therapeutic strategy to delay DN progression in animal models; however, the clinical trials should be performed to investigate the safety and efficacy of MSC transplantation in patients with DN [68]. Overall, stem cells could be used as an early diagnosis marker of DN and have a tendency to play much more important roles in the future.

### 5.4. Further Research about Traditional Chinese Medicine (TCM)

TCM has long histories in China and has emerged and influenced hundreds of thousands of people. Historically, conventional medicines, such as ACEI or ARB, are not able to totally prevent the development of DN [16]. Thus, there is an urgent need to find new effective agents to delay the progression of DN. TCM could produce prominent effects and there are several expert consensuses and recommendations for the treatment of CKD by improving proteinuria, such as Tripterygium and Emodin [69, 70]. Nowadays, an increasing understanding and popularity of TCM caused great interests in lots of diseases on its efficiency and mechanisms by the spring up of molecular biology, especially the application of Ultra Performance Liquid Chromatography (UPLC) and mass spectrometer which could analyze the active ingredients of TCM and manifest herbal medicine as western medicine. For the past few years, notoginsenoside R1 could retard DN by ameliorating podocyte adhesion through *α*3*β*1 integrin upregulation and astragaloside IV could inhibit podocyte apoptosis by downregulation of PERK-ATF4-CHOP pathway [71, 72]. Furthermore, there is a present study that aimed to evaluate the effect of ARB combined with Chinese formula Qidan Dihuang Grain (QDDHG) in improving proteinuria of patients of DN [73]. Yiqi Yangyin Huoxue Method is also a valid complementary and alternative therapy in the management of diabetic nephropathy, especially in improving UAER, serum creatinine, fasting blood glucose, and beta-2 microglobulin [74]. These findings strengthen the therapeutic rationale for TCM in the treatment of DN and also provide new insights into the development of novel drugs for the prevention of DN. However, the clinical applicability of TCM in human DN needs to be further investigated.

## 6. Controversy in Current Therapy of DN

It is a long time that the relationship between hypertension and DN has been the subject of controversy, and after diabetes mellitus, hypertension is the second most commonly reported etiology of ESRD in the United States Renal Data System, and hypertension treatment targets in patients with DN remain important clinical concerns [75]. Recent study demonstrated that the association between the renin gene polymorphism and the risk for developing DN in patients with type 1 diabetes may solve this problem in the future [76]. Angiotensin converting enzyme inhibitors (ACEIs) and angiotensin II receptor antagonists (ARB) are widely used in DN; however, blockage of the rennin-angiotensin system may not completely delay disease progression. Thus, there are high priorities to develop new and effective therapeutic approaches to prevent the progression of DN.

## 7. Conclusions

Despite the benefits derived from strict control of glucose and blood pressure, many patients continue to enter into ESRD. Thus, there is urgent need for improving mechanistic understanding of DN and then developing new and effective therapeutic approaches to delay the progression of DN. Many studies strengthen the therapeutic rationale for TCM in the treatment of DN. However, feasibility and safety of these therapeutic approaches and the clinical applicability of TCM in human DN need to be further investigated.

## Figures and Tables

**Table 1:** Studies about reported new future therapies of DN.

Study/year	Design/numbers	Race	Endpoints
Irannejad et al., 2016 [10]	Retrospective single-center analysis, serum nesfatin-1 in patients, included 44 adult patients with type 2 diabetes and microalbuminuria and 44 control patients with type 2 diabetes and normoalbuminuria	Asians	Peripheral nesfatin-1 levels are markedly elevated in patients with type 2 diabetes and microalbuminuria
Katayama et al., 2016 [11]	Prospective multicenter-randomized analysis, the efficacy and safety of seven once-daily oral doses of finerenone, included individuals: 96	Asians	Finerenone reduced albuminuria without adverse effects on serum potassium levels or renal function
Fouad et al., 2016 [12]	Retrospective single-center analysis, the relationship between serum uric acid and hypertension in DN, included individuals: 986	Caucasians	Serum uric acid level may identify and link with the onset of hypertension in DN
Machingura et al., 2017 [13]	Prospective cross-sectional analysis, prevalence of and factors associated with DN in Zimbabwe, included individuals: 344	Blacks	Prevalence of DN is higher in type 1 and type 2 diabetes mellitus patients than previously reported in Zimbabwe
Perkowska-Ptasinska et al., 2016 [14]	Retrospective multicenter analysis, biopsy based data from 14 renal centers in Poland, included individuals: 352	Caucasians	The relatively high prevalence of potentially curative kidney diseases of renal biopsy in these patients
Kaidonis et al., 2016 [15]	Prospective multicenter analysis, the single nucleotide polymorphism (SNP) rs2910164 residing within microRNA-146a (miR-146a) is associated with DN, included individuals: 890	Caucasians	Rs2910164 is significantly associated with microvascular complications DN
Li et al., 2015 [16]	Prospective multicenter randomized analysis, the additional benefit and safety of the Chinese herbal granule Tangshen Formula (TSF) in treating DN, included individuals: 180	Asians	TSF appears to be a safe therapeutic treatment for DN patients

## References

[1] Chopra B., Sureshkumar K. K. (2014). Co-stimulatory blockade with belatacept in kidney transplantation. *Expert Opinion on Biological Therapy*.

[2] Brenneman J., Hill J., Pullen S. (2016). Emerging therapeutics for the treatment of diabetic nephropathy. *Bioorganic and Medicinal Chemistry Letters*.

[3] Saran R., Li Y., Robinson B. (2015). US renal data system 2014 annual data report: epidemiology of kidney disease in the United States. *American Journal of Kidney Diseases*.

[4] Lopez-Vargas P. A., Tong A., Howell M., Craig J. C. (2015). Educational interventions for patients with CKD: a systematic review. *American Journal of Kidney Diseases*.

[5] Uhlig K., Berns J. S., Carville S. (2016). Recommendations for kidney disease guideline updating: a report by the KDIGO Methods Committee. *Kidney International*.

[6] De Boer I. H., Rue T. C., Hall Y. N., Heagerty P. J., Weiss N. S., Himmelfarb J. (2011). Temporal trends in the prevalence of diabetic kidney disease in the United States. *The Journal of the American Medical Association*.

[7] (2007). KDOQI clinical practice guidelines and clinical practice recommendations for diabetes and chronic kidney disease. *American Journal of Kidney Diseases*.

[8] Tuttle K. R., Bakris G. L., Bilous R. W. (2014). Diabetic kidney disease: a report from an ADA consensus conference. *Diabetes Care*.

[9] Ahn J. H., Yu J. H., Ko S.-H. (2014). Prevalence and determinants of diabetic nephropathy in Korea: Korea National Health and Nutrition examination survey. *Diabetes and Metabolism Journal*.

[10] Irannejad A., Ghajar A., Afarideh M. (2016). Association of peripheral nesfatin-1 with early stage diabetic nephropathy. *Pathophysiology*.

[11] Katayama S., Yamada D., Nakayama M. (2016). A randomized controlled study of finerenone versus placebo in Japanese patients with type 2 diabetes mellitus and diabetic nephropathy. *Journal of Diabetes and Its Complications*.

[12] Fouad M., Fathy H., Zidan A. (2016). Serum uric acid and its association with hypertension, early nephropathy and chronic kidney disease in type 2 diabetic patients. *Jornal Brasileiro de Nefrologia*.

[13] Machingura P. I., Chikwasha V., Okwanga P. N., Gomo E. (2017). Prevalence of and factors associated with nephropathy in diabetic patients attending an outpatient clinic in Harare, Zimbabwe. *The American Journal of Tropical Medicine and Hygiene*.

[14] Perkowska-Ptasinska A., Deborska-Materkowska D., Bartczak A. (2016). Kidney disease in the elderly: biopsy based data from 14 renal centers in Poland. *BMC Nephrology*.

[15] Kaidonis G., Gillies M. C., Abhary S. (2016). A single-nucleotide polymorphism in the MicroRNA-146a gene is associated with diabetic nephropathy and sight-threatening diabetic retinopathy in Caucasian patients. *Acta Diabetologica*.

[16] Li P., Chen Y., Liu J. (2015). Efficacy and safety of tangshen formula on patients with type 2 diabetic kidney disease: a multicenter double-blinded randomized placebo-controlled trial. *PLoS ONE*.

[17] American Diabetes Association (2013). Diagnosis and classification of diabetes mellitus. *Diabetes Care*.

[18] Yang W., Lu J., Weng J. (2010). Prevalence of diabetes among men and women in China. *The New England Journal of Medicine*.

[19] Wang L., Zhou M., Astell-Burt T. (2015). Geographical variation in diabetes prevalence and detection in China: multilevel spatial analysis of 98,058 adults. *Diabetes Care*.

[20] Zhang L., Wang F., Wang L. (2012). Prevalence of chronic kidney disease in China: a cross-sectional survey. *The Lancet*.

[21] Liu Z. (2013). Nephrology in China. *Nature Reviews Nephrology*.

[22] Dagogo-Jack S., Santiago J. V. (1997). Pathophysiology of Type 2 diabetes and modes of action of therapeutic interventions. *Archives of Internal Medicine*.

[23] Dagogo-Jack S. (2003). Ethnic disparities in type 2 diabetes: pathophysiology and implications for prevention and management. *Journal of the National Medical Association*.

[24] Stryker L. S. (2016). Modifying risk factors: strategies that work diabetes mellitus. *Journal of Arthroplasty*.

[25] Matsusaka T., Sandgren E., Shintani A. (2011). Podocyte injury damages other podocytes. *Journal of the American Society of Nephrology*.

[26] Barisoni L., Mundel P. (2003). Podocyte biology and the emerging understanding of podocyte diseases. *American Journal of Nephrology*.

[27] Greka A., Mundel P. (2012). Cell biology and pathology of podocytes. *Annual Review of Physiology*.

[28] Mundel P., Reiser J. (2010). Proteinuria: an enzymatic disease of the podocyte. *Kidney International*.

[29] Peti-Peterdi J., Sipos A. (2010). A high-powered view of the filtration barrier. *Journal of the American Society of Nephrology*.

[30] Bressler R., Johnson D. G. (1997). Pharmacological regulation of blood glucose levels in non-insulin- dependent diabetes mellitus. *Archives of Internal Medicine*.

[31] Harrison A. L., Shields N., Taylor N. F., Frawley H. C. (2016). Exercise improves glycaemic control in women diagnosed with gestational diabetes mellitus: a systematic review. *Journal of Physiotherapy*.

[32] Schorr S. G., Hammes H.-P., Müller U. A., Abholz H.-H., Landgraf R., Bertram B. (2016). The prevention and treatment of retinal complications in diabetes. *Deutsches Ärzteblatt International*.

[33] Fioretto P. (2006). Renal protection in diabetes: role of glycemic control. *Journal of the American Society of Nephrology*.

[34] Ismail-Beigi F., Craven T., Banerji M. A. (2010). Effect of intensive treatment of hyperglycaemia on microvascular outcomes in type 2 diabetes: an analysis of the ACCORD randomised trial. *The Lancet*.

[35] Pimenta W., Korytkowski M., Evron W. (1995). Pancreatic beta-cell dysfunction as the primary genetic lesion in NIDDM: evidence from studies in normal glucose-tolerant individuals with a first-degree NIDDM relative. *JAMA: The Journal of the American Medical Association*.

[36] Ba J., Han P., Yuan G. (2016). Randomized trial assessing the safety and efficacy of sitagliptin in Chinese patients with type 2 diabetes mellitus inadequately controlled on sulfonylurea alone or combined with metformin. *Journal of Diabetes*.

[37] Inzucchi S. E., Maggs D. G., Spollett G. R. (1998). Efficacy and metabolic effects of metformin and troglitazone in type II diabetes mellitus. *New England Journal of Medicine*.

[38] Cavaghan M. K., Ehrmann D. A., Byrne M. M., Polonsky K. S. (1997). Treatment with the oral antidiabetic agent troglitazone improves *β* cell responses to glucose in subjects with impaired glucose tolerance. *The Journal of Clinical Investigation*.

[39] Malaisse W. J., Lebrun P. (1990). Mechanisms of sulfonylurea-induced insulin release. *Diabetes Care*.

[40] Nelson T. Y., Gaines K. L., Rajan A. S., Berg M., Boyd III A. E. (1987). Increased cytosolic calcium. A signal for sulfonylurea-stimulated insulin release from beta cells. *Journal of Biological Chemistry*.

[41] Kolterman O. G., Gray R. S., Shapiro G., Scarlett J. A., Griffin J., Olefsky J. M. (1984). The acute and chronic effects of sulfonylurea therapy in type II diabetic subjects. *Diabetes*.

[42] Ravindran S., Kuruvilla V., Wilbur K., Munusamy S. (2017). Nephroprotective effects of metformin in diabetic nephropathy. *Journal of Cellular Physiology*.

[43] Gu S., Zeng Y., Yu D., Hu X., Dong H., Hu C. (2016). cost-effectiveness of saxagliptin versus acarbose as second-line therapy in type 2 diabetes in China. *PLOS ONE*.

[44] DeFronzo R. A., Abdul-Ghani M. (2011). Type 2 diabetes can be prevented with early pharmacological intervention. *Diabetes Care*.

[45] Eknoyan G., Hostetter T., Bakris G. L. (2003). Proteinuria and other markers of chronic kidney disease: a position statement of the National Kidney Foundation (NKF) and the National Institute of Diabetes and Digestive and Kidney Diseases (NIDDK). *American Journal of Kidney Diseases*.

[46] Futrakul N., Futrakul P. (2012). Therapeutic resistance to ACEI and ARB combination in macroalbuminuric diabetic nephropathy. *Clinical Nephrology*.

[47] Ren F., Tang L., Cai Y. (2015). Meta-analysis: the efficacy and safety of combined treatment with ARB and ACEI on diabetic nephropathy. *Renal Failure*.

[48] Bomback A. S., Kshirsagar A. V., Amamoo M. A., Klemmer P. J. (2008). Change in proteinuria after adding aldosterone blockers to ACE inhibitors or angiotensin receptor blockers in CKD: a systematic review. *American Journal of Kidney Diseases*.

[49] Chogtu B., Magazine R., Bairy K. L. (2015). Statin use and risk of diabetes mellitus. *World Journal of Diabetes*.

[50] Sirtori C. R. (2014). The pharmacology of statins. *Pharmacological Research*.

[51] Chogtu B., Magazine R., Bairy K. L. (2016). Response to comment on: statin use and risk of diabetes mellitus. *World Journal of Diabetes*.

[52] Sternlicht H., Bakris G. L. (2016). Management of hypertension in diabetic nephropathy: how low should we go?. *Blood Purification*.

[53] Bakris G. L., Weir M. R., Shanifar S. (2003). Effects of blood pressure level on progression of diabetic nephropathy: Results from The RENAAL Study. *Archives of Internal Medicine*.

[54] Schjoedt K. J., Rossing K., Juhl T. R. (2006). Beneficial impact of spironolactone on nephrotic range albuminuria in diabetic nephropathy. *Kidney International*.

[55] Zhou C., Lu F., Cao K., Xu D., Goltzman D., Miao D. (2008). Calcium-independent and 1,25(OH)2D3-dependent regulation of the renin-angiotensin system in 1*α*-hydroxylase knockout mice. *Kidney International*.

[56] Haussler M. R. (1986). Vitamin D receptors: nature and function. *Annual Review of Nutrition*.

[57] Yuan W., Pan W., Kong J. (2007). 1,25-Dihydroxyvitamin D3 suppresses renin gene transcription by blocking the activity of the cyclic AMP response element in the renin gene promoter. *Journal of Biological Chemistry*.

[58] Larsson T., Nisbeth U., Ljunggren Ö., Jüppner H., Jonsson K. B. (2003). Circulating concentration of FGF-23 increases as renal function declines in patients with chronic kidney disease, but does not change in response to variation in phosphate intake in healthy volunteers. *Kidney International*.

[59] Fligny C., Barton M., Tharaux P.-L. (2011). Endothelin and podocyte injury in chronic kidney disease. *Contributions to nephrology*.

[60] Baggio L. L., Drucker D. J. (2007). Biology of incretins: GLP-1 and GIP. *Gastroenterology*.

[61] Sousa M. M., Yan S. D., Stern D., Saraiva M. J. (2000). Interaction of the receptor for advanced glycation end products (RAGE) with transthyretin triggers nuclear transcription factor kB (NF-kB) activation. *Laboratory Investigation*.

[62] Wöbke T. K., Sorg B. L., Steinhilber D. (2014). Vitamin D in inflammatory diseases. *Frontiers in Physiology*.

[63] Liew C. G., Andrews P. W. (2008). Stem cell therapy to treat diabetes mellitus. *Review of Diabetic Studies*.

[64] Hamza A. H., Al-Bishri W. M., Damiati L. A., Ahmed H. H. (2016). Mesenchymal stem cells: a future experimental exploration for recession of diabetic nephropathy. *Renal Failure*.

[65] Peired A. J., Sisti A., Romagnani P. (2016). Renal cancer stem cells: characterization and targeted therapies. *Stem Cells International*.

[66] Baban B., Liu J. Y., Payne S., Abebe W., Yu J. C., Mozaffari M. S. (2016). Status of stem cells in diabetic nephropathy: predictive and preventive potentials. *EPMA Journal*.

[67] Hickson L. J., Eirin A., Lerman L. O. (2016). Challenges and opportunities for stem cell therapy in patients with chronic kidney disease. *Kidney International*.

[68] Ezquer M. E., Ezquer F. E., Arango-Rodríguez M. L., Conget P. A. (2012). MSC transplantation: a promising therapeutic strategy to manage the onset and progression of diabetic nephropathy. *Biological Research*.

[69] Wang J., Huang H., Liu P. (2006). Inhibition of phosphorylation of p38 MAPK involved in the protection of nephropathy by emodin in diabetic rats. *European Journal of Pharmacology*.

[70] Pei W. Y., Yang C. H., Zhang X. L. (2016). Effects and mechanism of Tripterygium wilfordii on chronic glomerulo nephritis. *Genetics and Molecular Research*.

[71] Gui D., Wei L., Jian G., Guo Y., Yang J., Wang N. (2014). Notoginsenoside R1 ameliorates podocyte adhesion under diabetic condition through *α*3*β*1 integrin upregulation in vitro and in vivo. *Cellular Physiology and Biochemistry*.

[72] Chen Y., Gui D., Chen J., He D., Luo Y., Wang N. (2014). Down-regulation of PERK-ATF4-CHOP pathway by astragaloside IV is associated with the inhibition of endoplasmic reticulum stress-induced podocyte apoptosis in diabetic rats. *Cellular Physiology and Biochemistry*.

[73] Xiang L., Jiang P., Zhou L. (2016). Additive effect of qidan dihuang grain, a traditional chinese medicine, and angiotensin receptor blockers on albuminuria levels in patients with diabetic nephropathy: a randomized, parallel-controlled trial. *Evidence-based Complementary and Alternative Medicine*.

[74] Ou J. Y., Huang D., Wu Y. S. (2016). A meta-analysis of randomized controlled trials of yiqi yangyin huoxue method in treating diabetic nephropathy. *Evidence-Based Complementary and Alternative Medicine*.

[75] Rahimi Z. (2016). The role of renin angiotensin aldosterone system genes in diabetic nephropathy. *Canadian Journal of Diabetes*.

[76] Lawes C. M., Hoorn S. V., Rodgers A. (2008). Global burden of blood-pressure-related disease, 2001. *The Lancet*.

